# Evidence of Reversible Bradycardia and Arrhythmias Caused by Immunogenic Proteins Secreted by *T. cruzi* in Isolated Rat Hearts

**DOI:** 10.1371/journal.pntd.0003512

**Published:** 2015-02-03

**Authors:** Héctor O. Rodríguez-Angulo, Jhoan Toro-Mendoza, Juan A. Marques, Juan L. Concepción, Rafael Bonfante-Cabarcas, Yoliver Higuerey, Luz E. Thomas, Leandro Balzano-Nogueira, José R. López, Alfredo Mijares

**Affiliations:** 1 Laboratorio de Fisiología de Parásitos, Centro de Biofísica y Bioquímica, Instituto Venezolano de Investigaciones Científicas, Caracas, Venezuela; 2 Centro de Estudios Interdisciplinarios de la Física. Instituto Venezolano de Investigaciones Científicas, Caracas, Venezuela; 3 Servicio de Cardiología, Instituto de Medicina Tropical, Universidad Central de Venezuela, Caracas, Venezuela; 4 Laboratorio de Enzimología de Parásitos, Departamento de Biología, Facultad de Ciencias, Universidad de Los Andes, Mérida, Venezuela; 5 Unidad de Investigaciones en Bioquímica, Decanato de Ciencias de la Salud, Universidad Centroccidental “Lisandro Alvarado”, Barquisimeto, Venezuela; 6 Servicio de Cultivos de Células y Tejidos, Centro de Biofísica y Bioquímica, Instituto Venezolano de Investigaciones Científicas, Caracas, Venezuela; 7 Laboratorio de Fisiología Molecular Centro de Biofísica y Bioquímica, Instituto Venezolano de Investigaciones Científicas, Caracas, Venezuela; 8 Unidad de Agronomía y Soberanía Agroalimentaria, Instituto de Estudios Avanzados (IDEA), Caracas, Venezuela; 9 Department of Molecular Biosciences, School of Veterinary Medicine, University of California, Davis, Davis, California, United States of America; Albert Einstein College of Medicine, UNITED STATES

## Abstract

**Rationale:**

Chagas cardiomyopathy, caused by the protozoan *Trypanosoma cruzi*, is characterized by alterations in intracellular ion, heart failure and arrhythmias. Arrhythmias have been related to sudden death, even in asymptomatic patients, and their molecular mechanisms have not been fully elucidated.

**Objective:**

The aim of this study is to demonstrate the effect of proteins secreted by *T. cruzi* on healthy, isolated beating rat heart model under a non-damage-inducing protocol.

**Methods and Results:**

We established a non-damage-inducing recirculation-reoxygenation model where ultrafiltrate fractions of conditioned medium control or conditioned infected medium were perfused at a standard flow rate and under partial oxygenation. Western blotting with chagasic patient serum was performed to determine the antigenicity of the conditioned infected medium fractions. We observed bradycardia, ventricular fibrillation and complete atrioventricular block in hearts during perfusion with >50 kDa conditioned infected culture medium. The preincubation of conditioned infected medium with chagasic serum abolished the bradycardia and arrhythmias. The proteins present in the conditioned infected culture medium of >50 kDa fractions were recognized by the chagasic patient sera associated with arrhythmias.

**Conclusions:**

These results suggest that proteins secreted by *T. cruzi* are involved in Chagas disease arrhythmias and may be a potential biomarker in chagasic patients.

## Author Summary

Chagas disease, caused by the parasite *Trypanosoma cruzi*, is an endemic disease of Latin-American countries, affecting an estimated 8 million people in 21 countries. It is spread by the bite of triatomine reduvid bug. Due to immigration towards non-endemic regions, the disease can spread and affect people around the world via blood transfusions. Infection usually occurs in childhood, and some patients may develop acute myocarditis; however, most remain asymptomatic for many years before chronic cardiac and/or gastrointestinal manifestations appear. Chagas disease is characterized by an acute phase, which is generally asymptomatic, or oligosymptomatic, an indeterminate phase, which may persist for several years, and a chronic phase in which dilated cardiomyopathy and arrhythmias are primarily observed and sudden death may occur. Once heart failure develops, death usually occurs within several years. In this work, we demonstrate the pathophysiological role of proteins secreted by *T*. *cruzi* on cardiac arrhythmias. The antigenicity of these fractions was tested by an immunological test using chagasic patients’ sera associated with arrhythmias. We showed that perfusion of the proteins secreted by *T*. *cruzi*, in an isolated beating rat heart model, induced cardiac arrhythmias such as bradycardia and complete atrioventricular block.

## Introduction

Chagas disease is an important public health problem in Latin America currently affecting an estimated 8 million people in 21 countries and spreading by human migration to a number of non-endemic regions [1]. The protozoan *Trypanosoma cruzi* is the etiologic agent of the disease in mammals; the parasite is transmitted by blood-sucking triatomine bugs, blood transfusions or trans-placentally [2]. This illness is characterized by an acute phase, which is generally asymptomatic, or oligosymptomatic, an indeterminate phase, which may persist for several years, and a chronic phase in which dilated cardiomyopathy and arrhythmias are primarily observed. Chagas cardiomyopathy has been attributed to an alteration in intracellular ions, an imbalance between adrenergic and cholinergic innervations, to cellular and humoral autoimmunity and to parasitic effects or micro-ischemic disturbances [3].

Cardiac arrhythmias are one of the most important alterations in Chagas heart disease and may be associated with sudden death [4]. The principal disorders reported are atrial, ventricular extrasystoles, intraventricular and/or AV conduction disturbances and primary ST-T wave changes [5]. Classically, arrhythmias have been linked to autonomic dysfunction [6], anti-adrenergic and anti-cholinergic autoantibodies [7] and to wall motion abnormalities [8]. Although, Chagas patients may present with arrhythmias and sudden death in the absence of ventricular dysfunction (known as the arrhythmogenic form) [9], the causes associated with nonstructural arrhythmias are poorly understood. Notably, ST and T abnormalities, ventricular and supraventricular arrhythmias and low voltage QRS have been reported in a recent acute outbreak characterized by high parasitemia [10], which suggests that the secreted proteins of the parasite may be involved in arrhythmia generation.

The interaction between the parasite and the host cell has gained attention in the pathophysiology of Chagas disease. Parasite surface proteins, such as mucins, trans-sialidases and mucin-associated proteins (MAPs), are adhesion factors involved in the invasion of the host cell [11]. Additionally, these proteins are able to increase the intracellular calcium concentration to facilitate the entry of the parasite [12,13]. Interestingly, a recent report described a calcium overload in the ventricular myocytes of chagasic patients [14] that may be related to parasite signaling and could be responsible for the arrhythmias observed in Chagas disease. However, there are few reports that have linked protein secretion by *T*. *cruzi* with arrhythmias [15]. Consequently, the aim this study is to demonstrate that proteins present in *T*. *cruzi*-conditioned medium are able to produce arrhythmias in an isolated beating rat heart model.

## Materials and Methods

### Ethics statement

Written consent from all patients involved in this study was obtained prior to processing the samples. The collection of serum samples from adult Chagas disease patients’ was approved by the Bioethics Committee for Human Research of the Universidad Centro Occidental Lisandro Alvarado, Barquisimeto, estado Lara, Venezuela (IVIC-DIR-0480/09). Data on human subjects was analyzed anonymously and clinical investigations have been conducted according to the Declaration of Helsinki.

For animal experimentation the project was also approved by the COBIANIM (IVIC-DIR-0376/1509/2014). COBIANIM is an advisory body of IVIC, with national reach, in regards to the ethical use of animals in research, in accordance with national and international standards. This committee oversees all research activities at IVIC, requiring the use of animals and wildlife to meet with Venezuelan law and universal ethical values. The Commission assessed the methodological, bioethical and legal aspects of this project (by resolution IVIC/Nro. 127/November, 4, 2009, according to the Código de Bioética y Bioseguridad, Ministerio del Poder Popular para Ciencia, Tecnología e Industrias Intermedias Fondo Nacional de Ciencia, Tecnología e Innovación, 2008 and, Guide Laboratory Animals for care and use, Eighth Edition, www.nap.edu, see details in http://www.ivic.gob.ve/cobianim/?mod=proyectos.php).

### Animal samples

Sprague Dawley IVIC female rats (300–400 g.) were obtained from the Bioterio Services at IVIC. The animals were allowed to acclimate for 2 weeks prior to the study. They were housed in a clean wire mesh cages (10 rats per cage) and maintained under standard laboratory condition of 12 hours natural light and 12 hours darkness at ambient room temperature. The rats were fed on pellets and water was made available *ad libitum*.

### Cell culture and conditioned media

Vero (African green monkey kidney cells, ATCC CCL-81; American Type Culture Collection, Rockville, Md.) were maintained at 37°C and 5% CO_2_ in complete Minimum Essential Medium (Mediatech, Herndon, Va.) containing 10% heat-inactivated fetal bovine serum (FBS; Gibco-BRL, Gaithersburg, Md), 20 mM HEPES, 2 mM L-glutamine, 1 mM sodium pyruvate, and 50 μg of gentamicin/ml (all Sigma-Aldrich, St Louis, MO). Confluent Vero cultures plated in a 75 cm^2^ Easy Flask were infected with 2x10^5^ EP strain trypomastigotes/ml. EP human strain at a rate of 2 parasites per cell. The EP strain of *T cruzi* was isolated from a fatal human case in 1967 as describe by Contreras et al [16]. Free parasites were removed after 24 hours and the complete medium was changed at this point to medium FBS-free. The fifth or sixth day post-infection the conditioned serum-free medium was collected. The criteria for harvesting of the conditioned infected medium (CMi) were that a minimum of 75% of the *Vero* cells should remain adhered and that at least 2.5 x 10^6^ trypomastigotes/ml should be present in the supernatant. The medium was centrifuged at 3000 x g for 10 minutes to separate the parasites, and the supernatant was subsequently filtered using a 0.2 μm membrane filter (Millipore, Billerica, MA, USA) before being stored at -20°C until use. Control medium (CMc) was collected from uninfected Vero cells cultured under the same conditions.

Cells were enzymatically detached with a 1:1 mixture of trypsin (0.25% v/v) and EDTA (0.25%), were lysed at 4°C with ten rounds of sonication for 20 s at power level 3 with a 550 Sonic Desmembrator (Fisher Scientific, Pittsburgh PA, USA). The suspension was centrifuged at 3000 x g for 10 minutes at 4°C to separate the cell debris, and the supernatant was subsequently filtered using a 0.2 μm membrane filter (Millipore, Billerica, MA, USA) before being stored at -20°C until use.

The supernatant fluid which had been decanted was concentrated by using low-protein binding membrane Diaflo (Millipore, AmiconCorp., Cambridge, Massachusetts) ultrafiltration cell operated in a cold room (4°C) at 50 psi. The Diaflo Model 50 ultrafiltration cell is provided with internal stirring and has a capacity of 40 ml. Supernatant from *T*. *cruzi*-infected cells (henceforth CMi) and those from uninfected Vero cells (henceforth CMc) SFV-free were passed through a ultrafiltration membranes. In each round, the fraction was concentrated to a volume of 10 ml (4x) and washed three times with milli-Q water to remove the lower molecular weight proteins. The pore size cutoff used were 300, 50, and 10 Kda, and the ultrafiltrates obtained were a) >300 Kda, b) <300 and > 50 Kda and c) <50 Kda [17].

After wash, each fraction was reconstituted to a final protein concentration of 5 μg/ml using Minimum Essential Medium (MEM; 1.8 mM CaCl_2_, 0.81 mM MgSO_4_, 5.33 mM KCl, 117.24 mM NaCl, 1.0 mM NaH_2_PO_4_, 5.56 mM D-Glucose, with L-Glutamine, Phenol Red and essential amino acids). The osmolalities of the reconstituted medium were measured using an osmometer (model Osmette A, Precision Instruments, Sudbury, MA) and were adjusted at 295–300 mOsm and pH to 7.4 with HCl. Finally, three concentrated fractions, corresponding to three molecular weight ranges, were obtained for the CMc and the CMi as follows: unfiltered, > 50 kDa and < 50 kDa.

### Isolated Langendorff hearts

**Fig 1:**
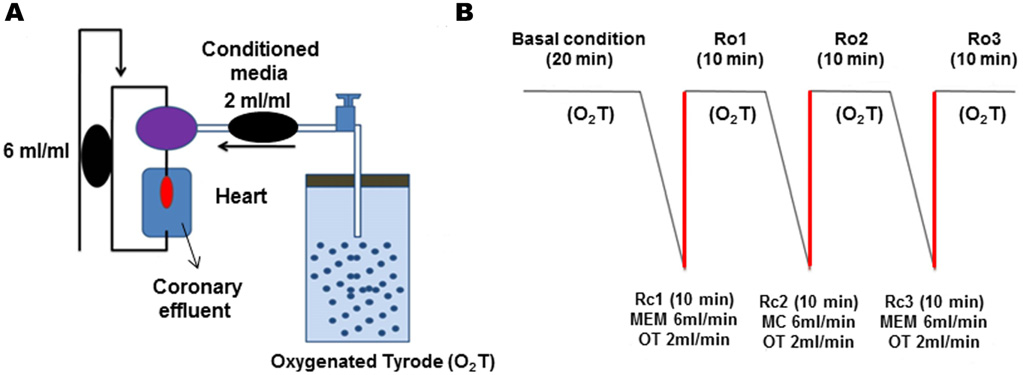
(A) Perfusion circuit with recirculation/ reoxygenation. The black ovals represent the two peristaltic pumps used to perfuse solutions. The magenta oval represents the device on which the cannula is inserted to perfuse the heart and red oval represents the heart. The black lines show the connections that allow the entry of oxygenated solution (O_2_T) in the heart. (B) Protocol of recirculation/reoxygenation. Rc: Recirculation and Ro: Reoxygenation. The red and diagonal black lines indicate the parallel perfused Conditioned medium or MEM.

For beating heart experiments, hearts were removed from adult female Sprague Dawley rats (weighing 300–400 g) previously anesthetized via intraperitoneal injection of pentobarbital (40 mg/kg). The isolated hearts were placed in cold Tyrode’s solution (25 mM sodium bicarbonate, 10 mM glucose, 116 mM NaCl, 3.3 mM KCl, 2.5 mM Ca_2_Cl, 1 mM MgSO_4_. and cannulated through the aorta. The hearts were perfused in a retrograde manner with warm Tyrode’s solution (37°C) for 30 minutes. The heart rate data were collected using the isolated beating heart system (PowerLab ADInstruments, Sydney, NSW, Australia). The presence of a sinus rhythm, a heart rate (HR) greater than 160 bpm, a perfusion pressure higher than 30 mmHg, and a flow rate of 2–6 ml/min were considered to be stable values for all experiments (Fig. 1, panel A).

The isolated hearts were perfused with conditioned media in accordance with a protocol consisting of three consecutive recirculation-reoxygenation cycles (Fig. 1, panel B). During the first recirculation stage, 5 mL of deoxygenated MEM was perfused in a closed circuit at a flow rate of 6 mL/min. To avoid cardiac cell damage due to anoxia, a 2 mL/min parallel flow of oxygenated Tyrode’s solution was added, whereby a standard flow rate of 6 mL/min was achieved with partial oxygenation. During the second recirculation stage, the hearts were perfused with: 1) Vero cell lysate or >50 kDa fraction (5 μg/mL) of 2) CMc (n = 5), 3) CMi (n = 8) and 4) CMi preincubated with serum samples of chagasic patients (n = 4), henceforth CMi+S. During the third stage, the hearts were perfused with MEM. Hearts in treatment received stabilization perfusion by one reoxygenation stage. The reoxygenation stage consisted of perfusing the heart with oxygenated Tyrode’s solution at a rate of 6 mL/min. All experiments were carried out under pression and perfusion controlled condition.

### Curve fitting

In this study, we used a Bolztmann equation for fitting to HR kinetics during recirculation-reoxygenation protocol as previously described [15] to model the I/R process.

### Chagas patients’ samples

A panel of Venezuelan Chagas patients’ samples of a sera collection compiled from 2002 to 2006 by the Laboratory of Parasites Physiology and the Cardiology Service of Instituto de Medicina Tropical, Universidad Central de Venezuela, was used for physiological assays. Inclusion criteria were Chagas disease diagnosed by two distinct Chagas tests, serological diagnosis was made with Cruzi ELISA kit as recommended by the manufacturer [18] and polymerase chain reaction (PCR) using a high conservation of DNA kinetoplast sequences (S35 5’-AAA TAA TGT ACG GG(T/G) GAG ATG CAT GA-32and S36 5’-GGG TTC GAT TGG GGT TGG TGT-3′) in *T*. *cruzi* allows detection of the parasite by means of an amplicon of 330 bp [19]. In this study, we used sera from Chagasic patients’ classified as Class II according to the New York Heart Association (NYHA) functional classification system: A serum of Class II Chagasic patients’ without arrhythmias was used as control in western blot analysis.

### Measurement of enzyme activity

To demonstrate preservation of myocardial function *ex vivo* during recirculation-oxygenation process, we determined aspartate aminotransferase (AST) activity by sampling the effusate. These activities were carried out in 10 μl aliquots of coronary effluent [20] in order to provide indication of cardiac damage. The samples were collected at the end of each recirculation-reoxygenation cycle. Each sample was immediately stored at -20°C until the analysis was performed. The enzyme activity was measured using a commercially available ELISA kit (Invelab, Caracas, Venezuela) in accordance with the manufacturer’s protocol.

### SDS—PAGE, immunoblotting and protein determination

SDS—PAGE was performed in 6–18% polyacrylamide gradient gels according to the method of Laemmli [21]. Proteins separated by SDS—PAGE were transferred to Immobilon-P filters (Millipore, USA). Fifteen micrograms of protein was loaded into each lane. Membranes were stained with Ponceau red to verify the transfer of the proteins. The membrane was incubated one hour with chagasic sera at a dilution 1/100 and washed three for five minutes in PBS and 0.05% Tween 20. The secondary antibody incubation was performed with peroxidase-conjugated goat anti-human immunoglobulin, diluted 1:5000. Immunoblots were developed by using diaminobenzidine (Sigma-Aldrich). Proteins were quantitatively assayed by the Lowry’s method as modified by Schacterle et al [22] with bovine serum albumin as standard.

### Statistical analysis

In order to quantify the relationships between continuous variables, QT, PR intervals and heart rate at the different experimental conditions, a Canonical Analysis of populations (CAP) were performed, then we performed a projection into a maximum information subspace which is known as canonical biplot analysis. CAP calculates the highest possible correlation between linear combinations of the values of studied variables, as within as between the a priori generated groups of individuals. Mean squared error (MSE), confidence interval and parameters functions was estimate on the parametric bootstrap methods for bias correction in linear mixed model (n = 250) [23]. Bootstraps were performed using InfoStat professional version (http://www.infostat.com.ar/) and the canonical biplot were carried out with MULTBIPLOT software [24].

## Results

### Effect of conditioned medium fractions on ECG recordings

**Fig 2:**
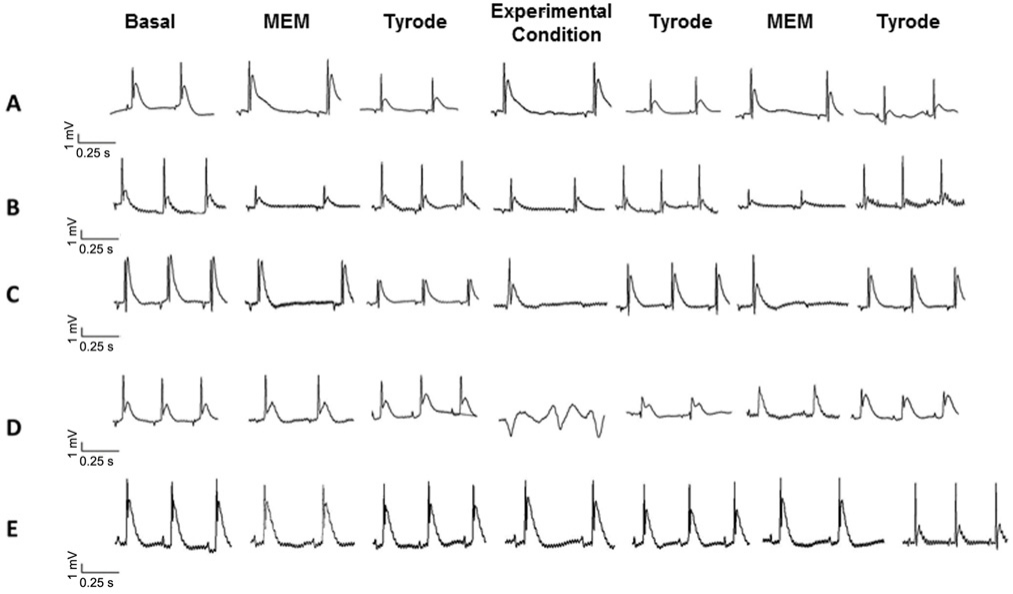
Representative electrocardiographic recordings made during the recirculation-reoxygenation protocol. The figure shows the recordings of the five hearts (A, B, C, D and E) carried out during stages of the perfusion process. Basal stage is the stabilization stage that represents the control condition (baseline) of each heart ECG. MEM/Tyrode’s (first recirculation-reoxygenation stage), Experimental Condition/Tyrode’s (second recirculation-reoxygenation stage), and a third perfusion of MEM/Tyrode’s similar to the first one. The perfusion in each experimental condition were A: Vero cell lysate; B: conditioned media (CM) control; C and D: CM infected; E: CM infected + serum. Panel E is a representative EGC of four chagasic patients’ sera tested independently. In this case, CM of Vero cells infected was pre-incubated with chagasic patient serum (1:10) for 30 min at 37°C and centrifuged at 3000 per g for 10 min before perfused. All experiments were carried out under pression and perfusion controlled conditions.

ECG recordings from hearts perfused with the different media are shown in Fig. 2. The panels A, B, C, D and E represents the ECG recordings of five hearts, basal recording corresponds to the internal control condition of each ECG heart. The hearts were subjected to three-recirculation and reoxygenation cycles (see fig. 1B). The recordings corresponding to the perfusion of hearts with either Vero cell lysate (Fig. 2A) or CMc (Fig 2B) did not show significant change at any stage of perfusion. In hearts perfused with CMi (Fig. 2C and 2D), a complete AV block with prolonged asystole was observed, together with an episode of ventricular fibrillation. In Fig. 2, panel E is a representative experiment of four co-incubations tested independently, of *T*. *cruzi* infected Vero cells conditioned media plus chagasic patients’ sera (CMi+S). Notably, it should be noted that chagasic patient’s sera preincubation abolished the observed effects when the hearts were perfused with >50 kDa proteins Cmi. Also, a partial reversion was observed in the 3^rd^ cycle, and a total reversion was achieved with Tyrode perfusion (Fig. 2E). We did not observe any abnormal cardiac conduction effects in the ECG recordings taken from hearts perfused with the <50 kDa fractions. It is known that the parasites secrete several bioactive lipids and glycolipids, thus lipid compounds were removed from conditioned medium and tested, and did not observe any abnormal cardiac conductions effect.

**Fig 3:**
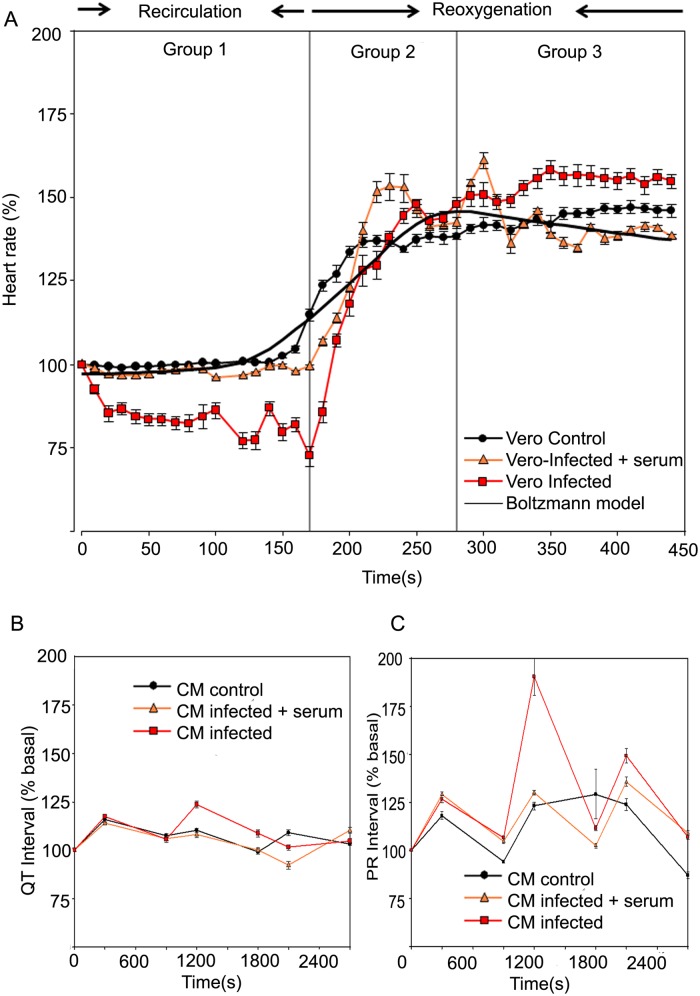
(A) Fixed models for heart rate (%) kinetics during the recirculation-reoxygenation protocol for the three different conditions studied. Percentage ± Confidence Interval95% were taken from the middle of the period of perfusion with conditioned medium (2.5 min) until the early recovery phase (5 min). These groups were comparing in three perfusion conditions (CM control, CM infected and CM infected + serum). In CM infected plus serum, medium was pre-incubated with chagasic patient serum (1:10) for 30 min at 37°C and centrifuged at 3000 per g for 10 min before perfused. Groups were defined as the heart rate in different frame times; Group 1, from 0 to 170 s, Group 2, from 171 to 280 s and, Group 3, higher than 280 s. The Boltzmann fitting is showed in solid line. (B) Fixed models for QT intervals during the recirculation-reoxygenation protocol for the three different conditions studied. (C) Fixed models for PR intervals during the recirculation-reoxygenation protocol for the three different conditions studied.

Fig. 3A shows the percentage of heart rate in three frame times in relation to the recirculation-reperfusion cycles. Three groups were defined by the heart rate in different frame times: Group 1, from 0 to 170 s, Group 2, from 171 to 280 s and, Group 3, higher than 280 s. The adjusted model represents the effect on heart rate kinetics for the three experimental conditions that resulted from perfusing hearts with the different fractions >50 kDa corresponding to CMc, CMi and CMi+S (S2 Table Rawdata Fig. 3A).

The adjusted linear mixed models (LMMs) shows that significant differences between conditions, times and its interaction (p≤0.05). The lowest heart rate percentage was found for the CMi and the highest for CMc. The average heart rate corresponds to 94.21 ± 1.16; 135.77 ± 1.18 and 150.61 ± 0.78 percent’s, for Groups 1, 2 and 3, respectively (see Fig. 3A). The comparison from CMi curve with Boltzmann model shows, in group 1, a significant decrease of initial HR parameter. These alterations, especially bradycardia, may be related to AV blockade observed in EGC (Fig. 2C). Remarkably, these changes were reversed during the reoxygenation stage of the same heart (Fig 2C and 2D) and/or by incubation of CMi+S (Figs. 2E and 3A).

Additionally, the Boltzmann analysis shows discrete changes of inflection times in hearts perfused with CMi and CMi+S, suggesting a HR recovery delayed effect during the early re-oxygenation stage probably related to immunogenic proteins (Fig 3, Groups 2 and 3). Also, we found difference between the initial HR values in the recirculation cycle in Cmi, this result suggests an involvement of secreted proteins in bradycardia. This bradycardia was independent of the QT interval over 450 s (Figs. 3A and 3B).

The Figs. 3B and 3C show the QT and PR segments estimated from EGC of isolated hearts perfused with CMc, CMi and CMi+S. This estimation was carried out over 2700 s. In both, QT and PR segments by LMMs adjusted analysis showed that condition CMi had the most variability observed compared with control condition (p≤0.05). The lowest QT interval was found for the CMi+S and the highest for CMi. The highest PR interval was observed was found for the CMi. This model demonstrates a sinusoidal behavior that cannot be associated with a tendency towards an increased PR interval (Fig. 3C).

### Enzymatic activity

**Fig 4:**
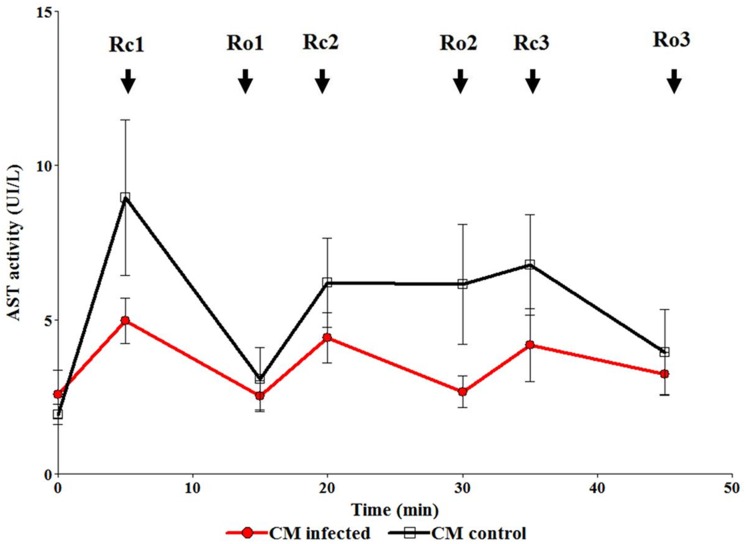
Determination of Aspartate aminotransferase activity (AST) in coronary effluent during the recirculation-reoxygenation cycles. Rc: recirculation cycle and Ro: reoxigenation cycle. The arrows identify the times at which the samples were taken of the recirculation-reoxigenation stages and are identified as Rc1 (recirculation 1), Rc2 (recirculation 2), Rc3 (recirculation 3), Rc1 (reoxygenation 1), Rc2 (reoxygenation 2) and Rc3 (reoxygenation 3). The graph shows the enzyme activity averages of the perfusato samples from hearts treated with CM control (n = 4) and CM infected (n = 5).

To evaluate cardiac cellular damage during the recirculation-reoxygenation protocol, we collected the media after each recirculation-reoxygenation cycle and measured AST activity (Fig. 4). The arrows identify the times at which the samples were taken. Rc1: recirculation 1; Rc2: recirculation 2; Rc3: recirculation 3; Ro1: reoxygenation 1; Ro2: reoxygenation 2; Ro3: reoxygenation 3. We observed that AST activity remained at basal levels during the entire protocol in hearts perfused with proteins >50 kDa CMi and CMc, which suggests the absence of cardiac cellular damage.

### Canonical biplot analysis

**Fig 5:**
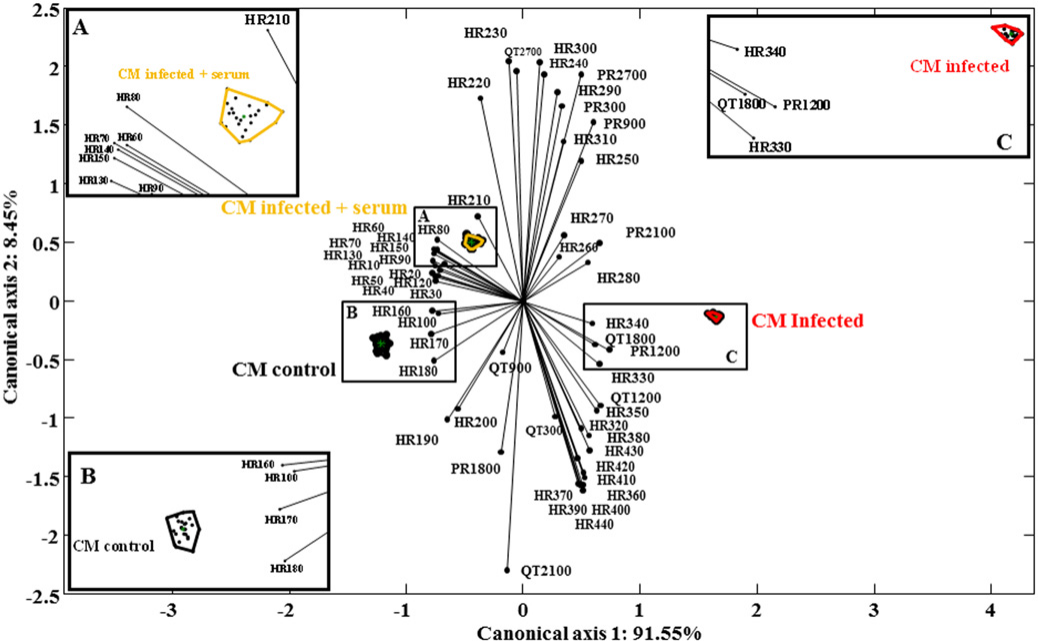
Canonical Biplot of the measured in three perfusion conditions. Comparing QT interval, PR interval, and heart rate in time. The axis 1 explain 91.55% and the axis 2 explain 8.45% of the variance with a Global contrast based on Wilk’s Lambda of 132.0474 and a p-value of 4.3475e-31. With the three (A, B & C) amplified regions we showed the convex hulls of the three groups and the calculated centroid. Due to scale differences between variables, each one were standardize separately. Analysis carried out with MULTBIPLOT software.

Fig. 5 describes the projection at different times of the variables and, the three conditions studied such as CMc, CMi and CMi+S, whose correlation was determined by Mahalanobis distance. The sum of percentages of two axis selected in the canonical biplot explain almost 100% of the variance with a very high quality of representation of the groups CMc (91.6%) and CMi (99.3%) in the canonical axis 1 and of CMi+S (56.3%) in the canonical axis 2 (S1 Table Rawdata canonic biplot).

The goodness of fit of the variables into the canonical subspace demonstrate that 16 of 55 variables were represented with quality over 80% (HR170:93.02, HR120: 91.2, HR180: 90.37, HR130: 89.79, HR160: 89.55, HR60: 88.96, HR150: 88.69, PR2700: 88.23, HR70: 86.77, HR50: 86.16, PR900: 85.41, HR30: 83.48, HR40: 83.43, HR80: 83.39, PR1200: 83.1, HR140: 82.18) 81.25% of the variables are related to the heart rate, and the rest are related to PR. Besides, 48 of 55 (87.27%) variables has a quality of representation over 50%, and 100% of the individuals were has a quality of representation above 85% in the canonical subspace.

### Immunogenicity of the proteins present in the conditioned medium

**Fig 6:**
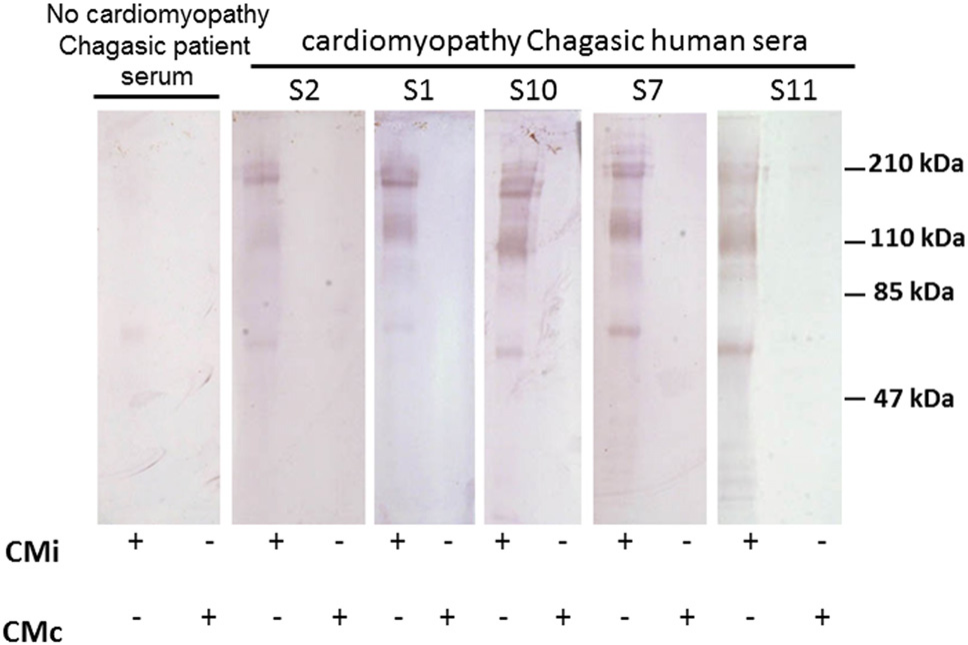
Western blotting analysis of conditioned medium. Protein fraction > 50 Kda obtained from CMi and CMc were resolved by gel electrophoresis and probed with sera samples (dilution 1:100) from seropositive class II chagasic patients that exhibited moderate symptoms of clinical disease. As controls it was assayed a no cardiomyopathy Chagasic patient serum.

To initiate characterization of the proteins in conditioned media that could be responsible for the observed effects on cardiac conduction, we performed western blotting with serum from chagasic patients’. As shown in Fig. 6, chagasic serum recognized various ultrafiltrate proteins. More antigenic proteins are in the range of molecular weight 50–200 kDa. In all cases, proteins in CMc were non-antigenic. Notably, chagasic serum obtained from patient without arrythmias did not reveal any bands.

## Discussion

In developing countries a proportion of chagasic patients die undiagnosed. Developing models to understand the pathophysiology of this disease and test new therapies are necessaries. The role of the secretome of the *T*. *cruzi* has been gaining attention. Traditional use of secretome protein in serodiagnosis, i.e. antigenic proteins detection by ELISA test called Trypomastigote Excreted-Secreted Antigens (TESA). There are currently no studies which associate *T*. *cruzi* secretome proteins as biomarkers for cardiac arrhythmias in Chagas cardiomyopathy. Recently, Wen et al (2012) [25] resolved the proteome signature of high and low abundance serum proteins in chagasic patients demonstrating the serum oxidative and inflammatory response profile, and serum detection of cardiac proteins parallels the pathologic events contributing to Chagas disease development. In this manuscript, we established a reproducible model using a recirculation-reoxygenation protocol that resembles the early interactions that occur between parasite secretome with cardiac cells. We found that CMi induces a biological effect on healthy, isolated beating rat heart model similar to those observed in Chagas patients. These effects may be related to the direct interaction of proteins into CM on heart cells. Some authors have demonstrated the persistence of the parasite in chronic lesions in patients [26], which reinforces the hypothesis that tissue damage is related to cellular parasitism *in vivo*. The detection of significant neuronal cell loss in the sympathetic and parasympathetic nervous systems of Chagas cases, in the absence of *T*. *cruzi in situ*, is the basis for the hypothesis of factors released from the parasite nest hidden somewhere in the host body, producing cell damage. [2]. Additionally, there is good *in vitro* and *in vivo* evidence for autoantibodies against neuroreceptors (beta-adrenergic and muscarinic) in Chagas disease [7,27]. In this study, we evaluated the role of the secretome fractions of *T*. *cruzi* co-cultured with Vero cells on cardiac arrhythmias using an isolated beating rat heart model. We observed bradycardia, ventricular fibrillation and complete atrioventricular block in hearts during perfusion with >50 kDa CMi. The antigenicity of the secreted proteins was tested by Western blotting using chagasic patient’s sera. The effects observed in this *in vitro* heart model are different from the results observed in autoimmunity studies [2], since we detected that immunogenic *T*. *cruzi*-secreted proteins was able alter cardiac function independent of a systemic immune response. It should be noted that chagasic patient’s sera preincubation abolished the observed effects when the hearts were perfused with >50 kDa proteins CMi, confirming the relationship between cardiovascular alterations, the immunogenic *T*. *cruzi*-secreted proteins and its correlation with arrhythmias in Chagas disease.

We have previously [15] demonstrated that cardiac damage can be estimated based on the amount of AST released into the coronary effluent. In the present study, we carried out serial AST enzymatic measurements as an internal control. The enzymatic activity remained stable through the recirculation-reoxygenation protocol, indicating lack of induced myocardial tissue damage.

The *T*. *cruzi* invasion has been linked to the interaction of parasite membrane glycoproteins with cellular ligands and their associated signaling pathways [28]. Several members of the *T*. *cruzi-*mucin family (TcMuc) have been linked to the process of invasion and to the increase in intracellular calcium concentration in particular. The secretion of MASP52, a member of the mucin associated protein (MASPs) family, has been associated with parasite attachment and with parasite invasion of Vero cells [29]. A recent report characterized the *T*. *cruzi* secretome obtained from medium conditioned by culturing the epimastigote and metacyclic trypomastigote forms under axenic conditions [30], in this study, it was demonstrated that proteins are shed in vesicles and that 3.8% of the secreted proteins are involved in parasite-cell interactions. The study identified the surface glycoprotein GP90, MASP52, trans-sialidase, and an 82 kDa glycoprotein, along with additional proteins, as being secreted by *T*. *Cruzi* (Table 1). However, these reports did not evaluate any pathophysiological role of this group of proteins.

**Table 1:** List of some proteins secreted by *T*. *cruzi*.

Protein	Accession number Uniprot	Gene name	References
Stage-specific surface glycoprotein GP82	Q9U7F3_TRYCR	gp82	[12, 13, 31]
Surface glycoprotein GP90	Q8M369_TRYCR	gp90	[30, 31]
Glycosomal glyceraldehyde phosphate (GP35/50)	Q5ZPH5_9TRYP	gGAPDH	[31]
mucin-associated surface protein (MASP52)	K4DV52_TRYCR	TCSYLVIO_006753	[29, 30]
glycoprotein 82 kDa	A7BI52_TRYCR	gp82 subfamily C3, gp82 subfamily C4	[30]
trans-sialidase	Q26964_TRYCR	TCTS-154	[30]
cathepsin-L	K4DV55_TRYCR C0J416_TRYBB	TCSYLVIO_006960	[32]

Ventricular arrhythmias in Chagas patients’ are related to calcium overload [14]. Accordingly the relationship between proteins secreted by the parasite and the regulation of intracellular calcium levels could provide insight into the arrhythmias observed in isolated beating hearts perfused with high molecular weight *T*. *cruzi* proteins. Previously, *T*. *cruzi* GP82, [13] GP90 and GP35/50 proteins [31] have been described as involved in both the modulation of calcium increase in the host cell and in determining the invasiveness of the parasite strain. Our group [15], demonstrated that *T*. *cruzi*-conditioned medium was able to increase the frequency of occurrence of tachyarrhythmia and cause a decrease in the heart rate during post-ischemic recovery. A novel approach recently reported by Elliott et al [32] showed that *Trypanosoma brucei* cathepsin-L supernatant disturbs the heart electrical activity, leading ventricular premature complex (which cause palpitations) and triggers arrhythmias in whole rat heart. In the present work, we observed a reversible ventricular fibrillation and a total AV block associated with bradycardia in a non-damage-inducing protocol, the effect was reversed by incubating the infected conditioned medium with chagasic patient’s serum, confirming that a direct interaction between the parasite secreted proteins and cardiomyocytes exist in the pathophysiology of Chagas cardiomyopathy. It is plausible that pro-arrhythmogenic proteins secreted or released by *T*. *cruzi* could act as enhancers causing the cardiac conduction system to cross an arrhythmic threshold in Chagas patients. This is the first report that implicates proteins secreted by *T*. *cruzi* with arrhythmias in an *ex-vivo* model.

We obtained a reproducible pattern of antigenic recognition of *T*. *cruzi*-secreted proteins by patient’s sera suggesting that immunogenic *T*. *cruzi*-secreted proteins are implicated with arrhythmias in Chagas disease. *T*. *cruzi* proteins could be used as virulence markers in the prognosis of the arrhythmias in Chagas patients.

To support the secretome proteins interaction a statistical analysis was carried out in order to quantify the relationships between continuous variables, QT and PR intervals and heart rates at the different experimental conditions (CMc, CMi and CMi+S). We decided to perform a Canonical Analysis of Populations (CAP). This methodology is used to project in a biplot simultaneously the structure of the *a priori* generated groups and the variables responsible for the separation between them. The CAP is used to obtain canonical axis that reflects the maximum separation between groups, not between individuals.

This statistical method has been used in other fields such as econometrics, and recently it has been used in biological systems was reported [33]. In this type of analysis there are two assumptions that should be tested, 1) the mean vectors of the groups must be significantly different. This assumption is evaluated by the study of global contrast based on Wilk’s Lambda (L) which turned out to be statistically significant 132.0474 with a p-value of 4.3475e-31. 2) the variances-covariances of the variables of the groups must be equal. The three variances-covariances matrices turned out to be singular which means that the variables have linear relation between them so it is possible to reduce the dimensionality of the system by eliminating variables. This was corroborated by the fact that with two dimensions we can explain almost 100% of the system variance, but it is important to remark that despite of the high quantity of redundant variables, they were ignored because of the use of Mahalanobis distance.

Canonical analysis of populations proved that 87.27% of the variables were represented with a quality above 50% and, 100% of the individuals were represented with a quality above 85%. This methodology permits us to elucidate two important aspects, first, we demonstrate that with just 16 variables we can explain over the 80% of the information and 13 of them are heart rate variables and the other three are PR variables. This means that the infection phenomena relative to the secretome proteins generated by the host-pathogen interaction could be successfully followed in time just by studying the heart rate. Besides not all time-points are necessary, just the mentioned above. Second, the group projections in the biplot analysis are in agreement with the results shown in Figs, 2 and 3, mainly in Fig. 2A which we can see the heart rate kinetics. We can infer that there is a significant recovery from bradycardia when the conditioned infected medium were mixed with chagasic patients serum, in other words the secretome proteins could be responsible for the heart dysfunction observed. We are already working on identifying the proteins of the secretome and how are their relationships with the dysfunction of the heart.

The contribution of this study was to evaluate, in an isolated heart model, the arrhythmogenic role of the parasite secretome proteins. This reproducible recirculation-reoxygenation model can be an useful system to investigate new drugs for the treatment of Chagas disease arrhythmias. The dissection and simplification of a complex system as constituting Chagas infection becomes necessary to understand and control the disease, in this sense, we wanted to contribute with a simplistic model which evaluates arrhythmogenic factors. This work represents a heuristic contribution to the study of arrhythmias produced by immunogenic proteins secreted by *Trypanosoma cruzi in vitro*. The findings of this study provide a glimpse into the role of this parasite’s secretome in the pathogenesis of the Chagas’ disease.

## Supporting Information

S1 Table Rawdata of fixed models for heart rate (%) kinetics during the recirculation-reoxygenation protocol for the three different conditions studied. (XLSX)

S2 Table Rawdata of Canonical Biplot analysis. (XLSX)

## References

[1] Rassi A Jr, Rassi A, Marin-Neto JA Chagas disease. The Lancet 375: 1388–1402. 10.1016/S0140-6736(10)60061-X 20399979

[2] Teixeira ARL, Hecht MM, Guimaro MC, Sousa AO, Nitz N (2011) Pathogenesis of Chagas’ Disease: Parasite Persistence and Autoimmunity. Clinical Microbiology Reviews 24: 592–630. 10.1128/CMR.00063-10 21734249 PMC3131057

[3] Higuchi MdL, Benvenuti LA, Martins Reis M, Metzger M (2003) Pathophysiology of the heart in Chagas’ disease: current status and new developments. Cardiovascular Research 60: 96–107. 14522411 10.1016/s0008-6363(03)00361-4

[4] Martinelli Filho M, De Siqueira SF, Moreira H, Fagundes A, Pedrosa A, et al (2000) Probability of occurrence of life-threatening ventricular arrhythmias in Chagas’ disease versus non-Chagas’ disease. Pacing Clin Electrophysiol 23: 1944–1946. 11139963 10.1111/j.1540-8159.2000.tb07058.x

[5] Elizari M (2002) Arrhythmias Associated with Chagas’ Heart Disease. Cardiac Electrophysiology Review 6: 115–119. 11984031 10.1023/a:1017911911178

[6] Thiers CA, Barbosa JL, Pereira BdB, Nascimento EMd, Nascimento JHd, et al (2012) Disfuncao autonomica e anticorpos contra receptores anti-m2 e anti-β1 em pacientes chagásicos. Arquivos Brasileiros de Cardiologia 99: 732–739. 22790404 10.1590/s0066-782x2012005000067

[7] Escobar AL, Fernandez-Gomez R, Peter JC, Mobini R, Hoebeke J, et al (2006) IgGs and Mabs against the beta2-adrenoreceptor block A-V conduction in mouse hearts: A possible role in the pathogenesis of ventricular arrhythmias. J Mol Cell Cardiol 40: 829–837. 16697002 10.1016/j.yjmcc.2006.03.430

[8] Barros ML, Ribeiro AL, Nunes MdC, Rocha MOdC (2011) Associação entre dissinergia miocárdica e arritmia ventricular na forma indeterminada da doença de Chagas. Revista da Sociedade Brasileira de Medicina Tropical 44: 213–216. 21503552 10.1590/s0037-86822011005000020

[9] Andrade JP, Marin-Neto JA, Paola AA, Vilas-Boas F, Oliveira GM, et al (2011) [I Latin American guidelines for the diagnosis and treatment of Chagas cardiomyopathy]. Arq Bras Cardiol 97: 1–48. 21952638

[10] Alarcón de Noya B, Díaz-Bello Z, Colmenares C, Ruiz-Guevara R, Mauriello L, et al (2010) Large Urban Outbreak of Orally Acquired Acute Chagas Disease at a School in Caracas, Venezuela. Journal of Infectious Diseases 201: 1308–1315. 10.1086/651608 20307205

[11] De Pablos LM, Osuna A (2012) Multigene Families in Trypanosoma cruzi and Their Role in Infectivity. Infection and Immunity 80: 2258–2264. 10.1128/IAI.06225-11 22431647 PMC3416482

[12] Yoshida N, Cortez M (2008) Trypanosoma cruzi: parasite and host cell signaling during the invasion process. Subcell Biochem 47: 82–91. 18512343 10.1007/978-0-387-78267-6_6

[13] Yoshida N, Favoreto S Jr., Ferreira AT, Manque PM (2000) Signal transduction induced in Trypanosoma cruzi metacyclic trypomastigotes during the invasion of mammalian cells. Braz J Med Biol Res 33: 269–278. 10719377 10.1590/s0100-879x2000000300003

[14] Lopez JR, Espinosa R, Landazuru P, Linares N, Allen P, et al (2011) [Dysfunction of diastolic [Ca(2)(+)] in cardiomyocytes isolated from chagasic patients]. Rev Esp Cardiol 64: 456–462. 10.1016/j.recesp.2011.01.008 21511385

[15] Rodriguez-Angulo H, Toro-Mendoza J, Marques J, Bonfante-Cabarcas R, Mijares A (2013) Induction of chagasic-like arrhythmias in the isolated beating hearts of healthy rats perfused with Trypanosoma cruzi-conditioned medium. Braz J Med Biol Res 46: 58–64. 23314340 10.1590/1414-431X20122409 PMC3854352

[16] Contreras VT, Araque W, Delgado VS (1994) Trypanosoma cruzi: metacyclogenesis in vitro—I. Changes in the properties of metacyclic trypomastigotes maintained in the laboratory by different methods. Memórias do Instituto Oswaldo Cruz 89: 253–259. 10.1016/j.nlm.2015.01.004 7885254

[17] Rhim JS, Williams LB RJ Jr., Turner HC (1969) Concentration by Diaflo ultrafiltration of murine leukemia and sarcoma viruses grown in tissue cultures. Cancer Res 29: 154–156. 4303443

[18] Berrizbeitia M, Concepcion JL, Carzola V, Rodríguez J, Cáceres A, et al (2012) Seroprevalence of T. cruzi infection in Canis familiaris, state of Sucre, Venezuela. 2012 33. 24652131

[19] Ramírez JD, Guhl F, Umezawa ES, Morillo CA, Rosas F, et al (2009) Evaluation of Adult Chronic Chagas’ Heart Disease Diagnosis by Molecular and Serological Methods. Journal of Clinical Microbiology 47: 3945–3951. 10.1128/JCM.01601-09 19846646 PMC2786654

[20] Jin C, Wu J, Watanabe M, Okada T, Iesaki T (2012) Mitochondrial K+ channels are involved in ischemic postconditioning in rat hearts. J Physiol Sci 62: 325–332. 10.1007/s12576-012-0206-y 22528048 PMC10717354

[21] Laemmli UK (1970) Cleavage of structural proteins during the assembly of the head of bacteriophage T4. Nature 227: 680–685. 5432063 10.1038/227680a0

[22] Schacterle GR, Pollack RL (1973) A simplified method for the quantitative assay of small amounts of protein in biologic material. Anal Biochem 51: 654–655. 4735559 10.1016/0003-2697(73)90523-x

[23] Kubokawa T, Nagashima B (2012) Parametric bootstrap methods for bias correction in linear mixed models. Journal of Multivariate Analysis 106: 1–16.

[24] JL V-V (2010) MULTBIPLOT: A package for Multivariate Analysis using Biplots. Departamento de Estadística Universidad de Salamanca (http://biplotusales/ClassicalBiplot/introductionhtml)

[25] Wen JJ, Zago MP, Nunez S, Gupta S, Burgos FN, et al (2012) Serum proteomic signature of human chagasic patients for the identification of novel potential protein biomarkers of disease. Mol Cell Proteomics 11: 435–452. 10.1074/mcp.M112.017640 22543060 PMC3412973

[26] Marcon GEB, Albuquerque DMd, Batista AM, Andrade PD, Almeida EA, et al (2011) Trypanosoma cruzi: parasite persistence in tissues in chronic chagasic Brazilian patients. Memórias do Instituto Oswaldo Cruz 106: 85–91. 10.1016/j.nlm.2015.01.004 21340361

[27] Costa PC, Fortes FS, Machado AB, Almeida NA, Olivares EL, et al (2000) Sera from chronic chagasic patients depress cardiac electrogenesis and conduction. Braz J Med Biol Res 33: 439–446. 10775309 10.1590/s0100-879x2000000400010

[28] Epting CL, Coates BM, Engman DM (2010) Molecular mechanisms of host cell invasion by Trypanosoma cruzi. Exp Parasitol 126: 283–291. 10.1016/j.exppara.2010.06.023 20599990 PMC3443968

[29] De Pablos LM, Gonzalez GG, Solano Parada J, Seco Hidalgo V, Diaz Lozano IM, et al (2011) Differential expression and characterization of a member of the mucin-associated surface protein family secreted by Trypanosoma cruzi. Infect Immun 79: 3993–4001. 10.1128/IAI.05329-11 21788387 PMC3187265

[30] Bayer-Santos E, Aguilar-Bonavides C, Rodrigues SP, Cordero EM, Marques AF, et al (2013) Proteomic analysis of Trypanosoma cruzi secretome: characterization of two populations of extracellular vesicles and soluble proteins. J Proteome Res 12: 883–897. 10.1021/pr300947g 23214914 PMC13615563

[31] Ruiz RC, Favoreto S ML Jr., Oshiro ME, Ferreira AT, et al (1998) Infectivity of Trypanosoma cruzi strains is associated with differential expression of surface glycoproteins with differential Ca2+ signalling activity. Biochem J 330 (Pt 1): 505–511. 9461549 10.1042/bj3300505 PMC1219166

[32] Elliott EB, McCarroll D, Hasumi H, Welsh CE, Panissidi AA, et al (2013) Trypanosoma brucei cathepsin-L increases arrhythmogenic sarcoplasmic reticulum-mediated calcium release in rat cardiomyocytes. Cardiovascular Research 100: 325–335. 10.1093/cvr/cvt187 23892734 PMC3797627

[33] Alkan B, Atakan C (2011) Use of canonical variate analysis biplot in examination of choline content data of some foods. International Journal of Food Sciences and Nutrition 62: 171–174. 10.3109/09637486.2010.523417 21271841

